# *Piper betle* (L): Recent Review of Antibacterial and Antifungal Properties, Safety Profiles, and Commercial Applications

**DOI:** 10.3390/molecules26082321

**Published:** 2021-04-16

**Authors:** Ni Made Dwi Mara Widyani Nayaka, Maria Malida Vernandes Sasadara, Dwi Arymbhi Sanjaya, Putu Era Sandhi Kusuma Yuda, Ni Luh Kade Arman Anita Dewi, Erna Cahyaningsih, Rika Hartati

**Affiliations:** 1 Department of Natural Medicine, Mahasaraswati University of Denpasar, Denpasar 80233, Indonesia; mmvsasadara@gmail.com (M.M.V.S.); or arymbhi@unmas.ac.id (D.A.S.); erasandhi@unmas.ac.id (P.E.S.K.Y.); armannita@unmas.ac.id (N.L.K.A.A.D.); or ernacahya@unmas.ac.id (E.C.); 2 Pharmaceutical Biology Department, Bandung Institute of Technology, Bandung 40132, Indonesia; rika@fa.itb.ac.id

**Keywords:** antibacterial, antifungal, betel leaves, *Piper betle*

## Abstract

*Piper betle* (L) is a popular medicinal plant in Asia. Plant leaves have been used as a traditional medicine to treat various health conditions. It is highly abundant and inexpensive, therefore promoting further research and industrialization development, including in the food and pharmaceutical industries. Articles published from 2010 to 2020 were reviewed in detail to show recent updates on the antibacterial and antifungal properties of betel leaves. This current review showed that betel leaves extract, essential oil, preparations, and isolates could inhibit microbial growth and kill various Gram-negative and Gram-positive bacteria as well as fungal species, including those that are multidrug-resistant and cause serious infectious diseases. *P. betle* leaves displayed high efficiency on Gram-negative bacteria such as *Escherichia coli* and *Pseudomonas aeruginosa*, Gram-positive bacteria such as *Staphylococcus aureus*, and *Candida albicans*. The ratio of MBC/MIC indicated bactericidal and bacteriostatic effects of *P. betle* leaves, while MFC/MIC values showed fungicidal and fungistatic effects. This review also provides a list of phytochemical compounds in betel leaves extracts and essential oils, safety profiles, and value-added products of betel leaves. Some studies also showed that the combination of betel leaves extract and essential oil with antibiotics (streptomycin, chloramphenicol and gentamicin) could provide potentiating antibacterial properties. Moreover, this review delivers a scientific resume for researchers in respected areas and manufacturers who want to develop betel leaves-based products.

## 1. Introduction

*Piper betle* (L) commonly known as betel vine belongs to the family Piperaceae. It is a popular medicinal plant in Asia. The leaf is the most widely used and studied part of the betel vine. There are chewing habit practices of betel leaves in many countries which are believed beneficial for avoiding bad breath, strengthening the gum, preserving the teeth, and stimulating the digestive system [1,2]. In traditional medicine practices, betel leaves are used for vaginal douching in Indonesia [3], as a gargle mouthwash in India and Thailand [4], and as a treatment for dental problems, headaches, arthritis, and joint pain in Malaysia [1]. In Srilanka, the betel leaf juice is used to treat skin ailments [5]. Additionally, its boiled leaves could be used as cough medicine, tonic, or astringent [2]. Traditional applications of betel leaves are related to their antibacterial and antifungal properties.

Over the past decades, antibacterial resistance has been threatening humans and has caused a global health crisis. Some bacterial strains are resistant to antibiotics such as vancomycin intermediate *Staphylococcus aureus* (VISA), vancomycin-resistant *Enterococcus* (VRE), methicillin-resistant *S. aureus* (MRSA), and extended spectrum β-lactamase (ESβL) enzyme producing Gram-negative bacteria, *Pseudomonas aeruginosa*, *Streptococcus pneumoniae*, *S. aureus*, and *Mycobacterium tuberculosis*, *Enterococcus faecium*, *Klebsiella pneumoniae*, *Acinetobacter baumannii*, and *Enterobacter* spp. [6,7]. Besides bacteria, fungi can also lead to infectious diseases. Approximately 300 fungal species on Earth are known to cause illnesses such as *Candida* spp. and dermatophytes [8,9]. Moreover, in the food industry, bacteria and fungi cause problems during product processing and storage. Food spoilage due to pathogen contamination is not only harmful to consumers but also brings heavy economic losses to manufacturers [10]. Therefore, research in this area continues to develop new safe and effective antimicrobial agents that could be applied in many related fields.

In this paper, a review of the literature was conducted to display recent studies (published in 2010–2020) on the antibacterial and antifungal properties of betel leaf extract (BLE), essential oil (BLEO), preparations, and isolates. In addition, the phytochemical constituents, safety profiles, and value-added products of betel leaves are also provided. Research on antibacterial and antifungal properties of betel leaves and their safety profiles have established their application as future active and additive ingredients in the pharmaceutical and food industries. Betel leaves are highly abundant and inexpensive, thus supporting their further development in manufacturing commercial products.

## 2. Phytochemicals in Betel Leaves

### 2.1. Betel Leaves Extract (BLE)

**Figure 1:**
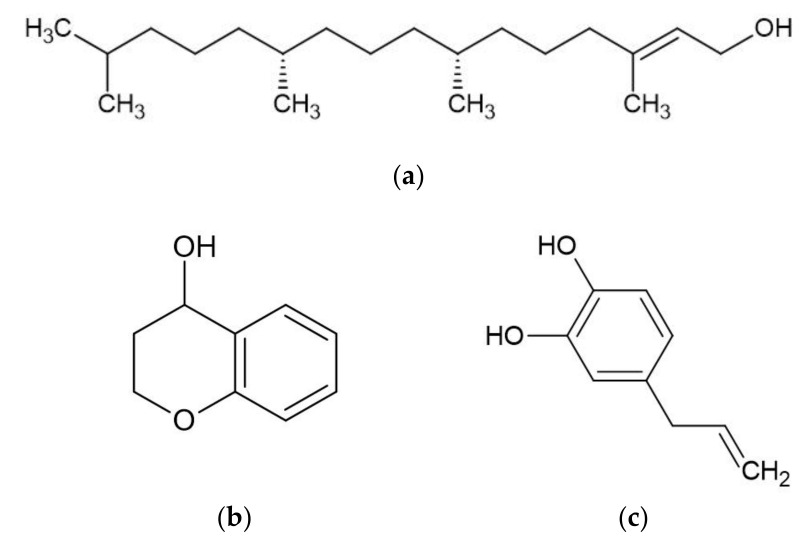

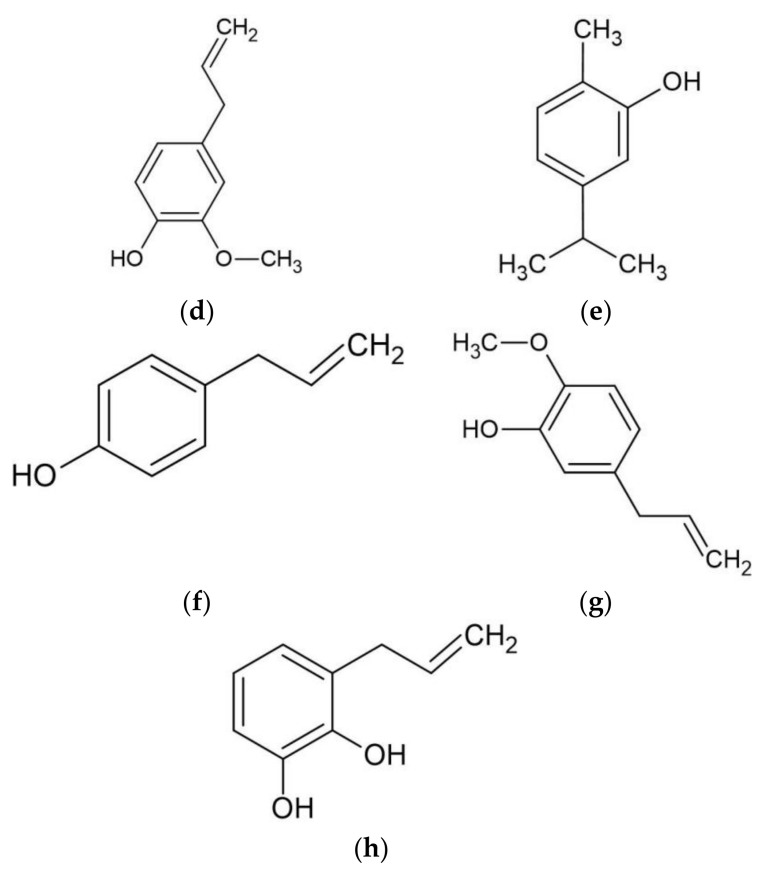
Major bioactive compounds in betel leaves extracts and essential oil. (**a**) phytol; (**b**) 4-chromanol; (**c**) hydroxychavicol; (**d**) eugenol; (**e**) carvacrol; (**f**) chavicol; (**g**) chavibetol; (**h**) allylpyrocatechols 1.

*Piper betle* contains numerous phytochemicals depending on its botanical origin and the solvent used for extraction. A preliminary phytochemical analysis of betel leaves from Malaysia showed that alkaloids, tannins, glycosides, reducing sugars, and saponins were found in the water extract of betel leaves [11]. Moreover, a study determined the total content of phenol, flavonoid, and tannin in water, ethanol, ethyl acetate, acetone, and dichloromethane extracts of betel leaves from Mauritius [12]. The highest total phenol, flavonoid, and tannin were found in the acetone, dichloromethane, and ethanol extracts, respectively. The sample of betel leaves collected from Tamilnadu, India is known to contain steroids, tannins, proteins, amino acids, flavonoids, terpenoids, mucilage, volatile oil, saponin, carbohydrates, and fixed oil, but an absence of alkaloids [13]. Furthermore, some studies have effectively isolated bioactive compounds from BLE (Figure 1) such as phytol, acyclic diterpene alcohol, 4-chromanol, hydroxychavicol or 4-allylpyrocatechol, and allylpyrocatechols 1 [14,15,16,17].

### 2.2. Betel Leaves Essential Oil (BLEO)

Betel leaves contain 0.15% to 0.2% essential oil which are classified as monoterpenes, sesquiterpenes, phenylpropanoids, and aldehydes (Table 1). The constituents of BLEO are strongly dependent on its botanical origin, age of the plant, and harvesting time. Various compounds of BLEO may affect its aroma, taste, and bioactivity [18]. GC-MS analysis of BLEO from different places in India showed that phenylpropanoid groups such as acetyl eugenol, eugenol, chavicol, and safrole were the major components [19]. Interestingly, Indian BLEO obtained from the Sagar Bangla cultivar contained chavicol, but not from the Magahi cultivar. The study also revealed that BLEO contained eugenol (40%) and a combination of carvacrol and chavicol (up to 40%) with chavibetol as a marker compound as depicted in Figure 1. Meanwhile, another study found additional main compounds including estragole, linalool, α-copaene, anethole, and caryophyllene α-terpinene, p-cymene, 1,8-cineole, β-caryophyllene, α-humulene, allyl pyrocatechol, allylcatechol, methyl eugenol, estragol (methyl chavicol), chavibetol, chavibetol acetate, safrol, 4-allyl-2-methoxy-phenolacetate, and 3-allyl-6-methoxyphenol [18,20,21].

## 3. Antibacterial Property of Betel Leaves

The extract, essential oil, preparation, and isolated compounds of betel leaves are effective against numerous Gram-negative (Table 2) and Gram-positive bacteria (Table 3). The bacteria tested included foodborne pathogens and other bacteria, including multidrug-resistant (MDR) bacteria that cause severe infectious diseases in humans. Most of the published research investigated the antibacterial activity of BLEs resulting from solvents with different polarities such as water, ethanol, ethyl acetate, acetone, and dichloromethane. Each extract contained diverse bioactive compounds which may affect their antibacterial activity [12,22]. The antibacterial tests of betel leaves were varied in methods and results, complicating the comparison between studies. Furthermore, the current review showed that the study of antibacterial activity of BLE was greater than that of BLEO.

A study showed that the ethanol extract of betel leaves was more effective than the water extract with greater inhibition zones. The ethanol extract at 50–100 µg/mL had the maximum inhibition zones (8.9–11.0 mm) on *E. coli* and moderate inhibition was observed on *P. aeruginosa* (<7.2 mm). Meanwhile, the water extract at 50 µg/mL did not actively inhibit bacterial growth [11]. Another investigation using the agar well diffusion method showed that the ethanol extract of betel leaves showed greater inhibition zones on Gram-negative than Gram-positive bacteria [17]. A study demonstrated the antibacterial effect of five types of BLE resulting from different polarities of solvents. Among these extracts, acetone and ethyl acetate extracts demonstrated the most remarkable activity against the six bacteria tested, with *S. aureus* being the most susceptible one. Moreover, the antibacterial property of BLEs was related to their phenol and flavonoid contents [12].

Other than the inhibition zone, the antibacterial activity was also presented as minimum inhibitory concentration (MIC) and minimum bactericidal concentration (MBC). MIC is defined as the lowest concentration of samples that inhibits microbial growth. Meanwhile, MBC is the lowest sample concentration at which 99.9% of the bacteria are killed [23]. For easier comparison, the MIC and MBC values from published articles were recalculated from μg/mL, mg/mL, and µg/µL to percentage (*w*/*v* or *v*/*v*).

The most frequently studied Gram-negative bacteria were laboratory strains of *E. coli* and *P. aeruginosa* with MIC range from 0.03 to 0.4% and 0.05–0.4%, respectively [12,12,13,14,15,16,17,18,19,20,21,22,23,24,25,26,27,28]. Meanwhile, the lowest MIC (0.0156%) among Gram-negative bacteria was documented for clinical isolates of *P. aeruginosa* MβL(+) 3, *A. baumannii* MβL(+) 2, and *P. aeruginosa* MβL(+) 3 [29]. Additionally, *S. aureus* was the most commonly used Gram-negative bacteria to screen the antibacterial effect of betel leaves with MIC range from 0.00025 to 0.15% [12,24,25,26,27,28]. The lowest MIC among Gram-positive bacteria was recorded for an oral pathogen *Streptococcus gordonii* DMST 38731 (0.00005%) [30].

In this review, the MBC/MIC ratio was also measured to show the bacteriostatic and bactericidal effects of betel leaves. If the ratio is ≤2, the samples are considered to be bactericidal agents. The bacteriostatic mode of action is reflected when the ratio is ≥4 [31]. BLEO showed only a bactericidal effect and BLE was found to be bacteriostatic and bactericidal. The bactericidal action was reported against Gram-negative and Gram-positive bacteria, including those classified as MDR bacteria such as ESβL-producing *Enterobacteriaceae*, carbapenem-resistant *Enterobacteriaceae* (CRE), Metallo-β-lactamase (MβL)-producing *P. aeruginosa* and *A. baumannii*, MRSA, and VRE. On the other hand, a bacteriostatic effect was only observed against Gram-positive bacteria *Streptococcus gordonii*.

A previous study proved the promising antibacterial effect of BLE against oral pathogens including Gram-positive cariogenic bacteria and Gram-negative periodontal pathogenic bacteria. The study also found that 4-chromanol was the compound responsible for the antibacterial and antibiofilm properties of BLE [17]. Another study discovered the ability of BLE to control biofilm formation of *Vibrio harveyi* [32]. The antibacterial effect of BLE was dose-dependent. BLE was also found to be effective in reducing biofilm formation and extracellular polymeric substance production caused by *P. aeruginosa* and bacterial consortium without increasing the selective pressure for the growth of microorganisms [33]. Additionally, the ethyl acetate extract of betel leaves could act as antibiofilm agents against the nosocomial pathogen *Serratia marcescens* through the inhibition of quorum sensing mediated virulence factors production such as protease and lipase [16].

*P. betle* showed an outstanding antibacterial activity compared with other plants. The previous study compared the antibacterial activity of the ethanol extract of 12 plants from the Philippines, namely *Cassia alata*, *Centella asiatica*, *Curcuma longa*, *Psidium guajava*, *Piper betle*, *Vitex negundo*, *Mitrephora lanotan*, *Moringa oleifera*, *Phyllanthus niruri*, *Tinospora rumphii*, and *Zingiber officinale,* against clinical isolate of MRSA, VRE, ESβL-producing *Enterobacteriaceae*, CRE, and MβL-producing *P. aeruginosa* and *A. baumannii*. *Piper betle* was the only plant that showed potent bactericidal activity against all the bacteria tested with an MBC/MIC ratio between 1 to 2 [27]. Another investigation exhibited the higher antibacterial activity of ethanol extract from betel leaves compared to other medicinal plants such as *Andrographis paniculata*, *Momordica charantia*, *Phyllantus emblica*, *Psidium guajava*, and *Sesbania grandiflora*. The study also revealed that ethyl acetate fraction showed the strongest antimicrobial activity compared to hexane and ethanol fractions and crude ethanol extract. Further, the ethyl acetate fraction showed higher inhibition zones and MIC against *Streptococcus gordonii* than the positive control (chlorhexidine solution) [30].

It is noteworthy that natural products could provide additive antimicrobial activity and modify antibiotic resistance when combining with conventional antibiotics [34]. The synergistic effect was found in a combination of ethyl acetate or acetone extract of betel leaves and streptomycin and chloramphenicol against *P. aeruginosa*, *S. aureus*, *Propionibacterium acnes*, *Staphylococcus epidermidis*, and *Streptococcus pyogenes*. The highest synergy was observed when the acetone extract and chloramphenicol combination (70:30) was used against *P. aeruginosa*. However, there was no correlation between phytochemical content and the synergistic effect which indicated a different mechanism of action [12]. A study also revealed a potentiating effect of BLEO and gentamicin against *Escherichia coli* and *S. epidermidis* [24]. These results should be further confirmed to assure the effectiveness of betel leaves as an antibacterial potentiating agent.

Some research evaluated the antibacterial activity of the BLE or BLEO based preparation against different pathogens. The antibacterial activity of silver-BLE nanoparticles was found to be similar to standard drug (norfloxacin) against *S. aureus*. The nanoparticles also exhibited a bacteriostatic effect on *Salmonella typhi*, *E. coli*, and *P. aeruginosa*. Moreover, the previous study concluded that Gram-positive bacteria are more susceptible to silver-BLE nanoparticles rather than Gram-negative bacteria [25]. Another study also developed the green synthesis of CaO nanoparticles using the water extract of betel leaves. It showed maximum and minimum activity against *E. coli* and *Streptococcus mutans*, respectively [28]. Additionally, BLEO based nanoemulsion was observed to be effective against five strains of foodborne pathogens and can be used as a promising natural antibacterial agent in the food system [26].

The isolated phenolic compound of BLE, namely hydroxychavicol or allylpyrocatechols, were tested against *Streptococcus sanguinis*, a Gram-positive bacterium that contributes to caries [15]. The compound was a moderate antibacterial agent that functioned by blocking MurA that causes bacterial cell wall disruption. The result exhibited the potential of betel leaves as an alternative effective and efficient treatment for mechanical plaque removal through inhibition of bacterial growth. The isolate could also kill *Streptococcus intermedius* and *S. mutans* by a similar mechanism mentioned above. The study showed that the killing kinetic of 4-allylpyrocatechol was dose and pathogen dependent [35]. The overgrowth of these bacteria develops many serious oral infections and are the major cause of caries, gingivitis, and chronic periodontitis [36].

## 4. Antifungal Properties of Betel Leaves

Numerous methods have been applied to test the antifungal properties of betel leaves including solid dilution, broth dilution, micro-dilution, well diffusion, and solid diffusion assays, resulting in minimum inhibitory concentration (MIC), minimum fungicidal concentration (MFC), and inhibition zones (Table 4). Similar to antibacterial activity, recalculation of MIC and MFC, and measurement of MFC/MIC ratio to determine fungicidal and fungistatic effects, were also conducted. *Candica albicans* was the most screened fungal species with MIC ranging from 0.01% to 0.07% [2,24,30,35,38,39] The fungicidal effects of BLE and BLEO against various fungal species including *Aspergillus flavus*, *Aspergillus fumigatus*, *Aspergillus niger*, *Aspergillus parasiticus*, *C. albicans*, *Candida glabrata*, *Candida krusei*, *Candida neoformans*, *Candida parapsilosis*, *Candida tropicalis*, *Epidermophyton floccosum*, *Trichophyton mentagrophytes, Trichophyton rubrum*, *Microsporum canis*, and *Microsporum gypseum* [24,30,38,40]. Meanwhile, the fungistatic effect was only recorded from hexane and ethyl acetate extract of betel leaves against *C. albicans* [30], and its isolate, hyroxychavicol, against *C. krusei* [38]. A few of these species can contaminate food and spread aflatoxin, which is harmful to humans [18,41]. Other fungal species are clinically significant human pathogens that cause dental disorders and dermatophyte infections [2,14,35,40].

Ethanol and ethyl acetate extracts of betel leaves were found to be effective against *C. albicans* isolated from oral thrush patients. The ethyl acetate extract demonstrated the highest inhibition zone compared to extracts from another plant (*Ocimum sanctum*) and a standard drug (fluconazole) [39]. Other studies have also demonstrated the greater antifungal activity of ethyl acetate extract compared to hexane and ethanol extracts of betel leaves [10,30]. The killing kinetic study revealed that the fungistatic activity of the ethyl acetate extract was concentration-dependent. Furthermore, other research showed the anticandidal action of water extract from betel leaves. This effect was possibly related to its ability to reduce the cell surface hydrophobicity of several Candida species. Adhesion of fungal species and host tissues is crucial for fungal virulence, especially for successful colonization and infection. Hydrophobic domains in fungal surface proteins which consist of non-polar amino acids are a major factor involved in fungal adhesion. Thus a deviation in hydrophobic affinity produced by *P. betle* extract may influence the adherence mechanism of the fungal cell [42].

Some research investigated the antifungal activity of BLEO. A study showed that antifungal and aflatoxin suppressor actions of BLEO are related to its main components such as eugenol [18,40]. Eugenol contains a hydroxyl group that could form hydrogen bonds with the active site on fungal enzymes that are responsible for aflatoxin secretion and later causes denaturation [43]. Eugenol was also reported to induce fungal morphological abnormalities by changing or disrupting fungal cell wall structure, increasing cell membrane fluidity and permeability, and interfering with important regulator function [44]. Furthermore, docking simulation of eugenol acetate and chavicol acetate in BLEO showed strong interaction to amino acid constructing fungal protein structures, which is predicted to cause metabolic reduction and biomass breaking down, thus reducing fungal virulence [45].

The superior antifungal property of BLEO compared with essential oils from other Mauritius plants such as Psiadia argute, Psiadia terebinthina, Pimenta dioica, Salvia officinalis, Laurus nobilis, Rosmarinus officinalis, Cinnamomum zeylanicum, and Schinus terebinthifolius has been proven. The study revealed that BLEO was the strongest fungicidal agent with the lowest MIC against all the ATCC strains and clinical isolates fungi tested [24]. A formula of BLEO based microemulsion showed tremendous fungi toxic activity against a selected mold in raw apple juice at low concentration (<0.5 µL/mL). Meanwhile, spore inactivation of A. flavus and P. expansum by BLEO was found at a greater concentration (15 µL/mL) [41].

Hydroxychavicol or 4-allylpyrocatechol isolated from betel leaves was also reported to be effective against various fungi species. The compound could entirely kill *C. albicans* at a minimum concentration (400 μg/mL) [35]. The killing ability of hydroxychavicol against *C. albicans* and *C. glabrata* was dose-dependent. Hydrochavicol demonstrated fungicidal effects against other clinical isolates fungi, with the MICs ranging from 7.81 to 62.5 μg/mL for dermatophytes, 15.62 to 500 μg/mL for yeasts, and 125 to 500 μg/mL for *Aspergillus* species, while the MFCs were found to be equal or two-fold higher than the MICs [38]. Moreover, it could prevent biofilm formation and promote biofilm eradication [35,38]. The development of a biofilm, which is a network of microbial cells tightly adsorbed at the mucosal surface, is linked to a severe infection [46].

## 5. Safety Profiles of Betel Leaves

An acute toxicity study in both male and female ICR mice showed the safety of the methanol extract of betel leaves orally. The median lethal dose (LD50) of the extract was higher than 5000 mg/kg body weight [47]. There was also an evaluation of oral acute and sub-acute toxicity (28 days) and genotoxicity of an herbal formulation containing betel leaves alcoholic extract in rats and cellular models. This study revealed the absence of major adverse reactions [48]. Moreover, betel leaves were considered safe in terms of hematotoxicity, hepatotoxicity, genotoxicity, weights of organs, gross morphology, stress, or aversive behaviors in rats [49]. Another study discovered the nontoxicity of the ethanol extract of betel leaves on normal human dermal fibroblasts (HDFn) [29].

## 6. Commercial Application of Betel Leaves

There are some available commercial products containing betel leaves such as dietary supplements, mouthwash, medicinal products, and cosmetic and personal care goods including shampoo, soap, face cream, antiseptic lotions, toothpaste, and perfumes [50]. Current antimicrobial studies of betel leaves were focusing on oral pathogens, MDR Gram-negative and Gram-positive bacteria, and dermatophytes [17,29,30,38]. Thus, future development of medicinal products from betel leaves could be useful for preventing oral diseases, curing dermatophyte infections, and for the treatment and management of other infectious diseases. Additionally, a study has developed a simple, safe, cost-effective, and eco-friendly preparation of silver nanoparticles with polyaniline coating using water extract of betel leaves. The nanoparticles showed potential antibacterial properties and could be further studied in various applications such as medical devices and pharmaceutical and biomedical industries [25].

In the food industry, essential oil is a promising food additive to protect and enhance the shelf life of products during processing and storage. BLEO is an ideal food preservative agent due to its antifungal and antioxidant properties [18]. Many experiments have investigated the antimicrobial properties of BLEO against foodborne pathogens [18,26,41]. Moreover, BLEO is not only beneficial to prevent spoilage of food products but also guarantees their safety for consumer health especially due to the ability of BLEO to suppress aflatoxin production. Aflatoxin, a mycotoxin from *A. flavus*, is an example of fungal contamination in food products. The toxin is known to be hepatocarcinogenic, teratogenic, mutagenic, and immunosuppressive. An investigation revealed that BLEO in apple juice could deactivate spores or inhibit spore germination which is required to limit fungal infection and mycotoxin production [41]. Further research on the overall acceptability of sensory aspects of the essential oil-treated foodstuffs is necessary to avoid market failure of the product [51].

## 7. Conclusions and Outlook

The antibacterial and antifungal properties and safety profiles of betel leaves firmly support their application in the development of various products, especially in the food and pharmaceutical industries. The utilization of betel leaves in producing modern-commercial goods could increase the economy of local farmers, specifically in Asia. A good agricultural process should be applied to the farm to yield standardized raw material and should be followed by a good manufacturing process in industries to form high-quality final products. Additionally, clinical studies should be conducted to support the use of betel leaves in medical fields. Researcher, government, and manufacturer collaboration could facilitate this necessary task.

## Figures and Tables

**Table 1:** List of phytochemicals identified from betel leaf essential oil

Classification	Compounds	Classification	Compounds
**Monoterpenes**	α-Thujene α-Pinene Camphene Sabinene Myrcene α-Terpinene β-Phellandrene 1,8-Cineole/Eucalyptol (E)-β-Ocimene γ-Terpinene Terpinolene Linalool Terpinen-4-ol α-Terpineol L-limonene Linalyl acetate	**Sesquiterpenes**	δ-Elemene α-Copaene β-Elemene E-β-Caryophyllene β-Copaene γ-Elemene Aromadendrene α-Humulene γ-Muurolene Germacrene D Germacrene B β-Selinene α-Selinene Bicyclogermacrene α-Muurolene cis-β-Guaiene δ-Cadinene or δ-amorphene Palustrol Spathulenol Caryophyllene oxide Globulol Viridiflorol Cubenol α-Cadinol Ledene α-amorphene Cubebene
**Phenylpropanoids**	Estragole/Methyl chavicol Chavicol Anethole/Isoestragole Safrole Chavicol acetate Eugenol Methyl eugenol Acetyl eugenol Phenyl acetaldehyde	**Aldehydes**	Undecanal Phenyl acetaldehyde

**Table 2:** *Piper betle* against Gram-negative bacteria.

**Extract/Preparation (Unit for Activities)**	**Method**	**Bacteria Species**	**Activities**		**Recalculated (%)**		**MBC/MIC**	**Inhibition Zone (mm)**	**Reference**
			**MIC**	**MBC**	**MIC**	**MBC**			
Ethanol	Agar well diffusion	*Pseudomenas aeruginosa*	-	-	-	-	-	6.7–7.2	[11]
		*Escherichia coli*	-	-	-	-	-	8.9–11.0	
Water	Agar well diffusion	*Pseudomenas aerugiaounosa*	-	-	-	-	-	7.2	[11]
		*Escherichia coli*	-	-	-	-	-	8.5	
Ethanol ( µg/mL)	Disk diffusion	*Escherichia coli* ATCC 25922	625	625	0.0625	0.0625	1 *	16	[27]
		*Klebsiella pneumoniae* ATCC BAA-1705	1250	1250	0.125	0.125	1 *	17	
		*Pseudomenas aeruginosa* ATCC 27853	625	625	0.0625	0.0625	1 *	17	
		MβL, *Pseudomenas aeruginosa* (CI)	312	312	0.0312	0.0312	1 *	17	
		MβL, *Acinetobacter baumannii* (CI)	625	625	0.0625	0.0625	1 *	23	
		ESβL, *Escherichia coli* (CI)	312	625	0.0312	0.0625	2 *	20	
		ESβL, *Klebsiella pneumoniae* (CI)	625	625	0.0625	0.0625	1 *	20	
		CRE, *Klebsiella pneumoniae* (CI)	312	625	0.0312	0.0625	2 *	21	
Ethyl acetate (µg/µL)	Micro dilution	*Escherichia coli* ATCC 25922	4.00	-	0.4	-	-	-	[12]
		*Pseudomenas aeruginosa* ATCC 27853	4.00	-	0.4	-	-	-	
Acetone (µg/µL)		*Escherichia coli* ATCC 25922	4.00	-	0.4	-	-	-	[12]
		*Pseudomenas aeruginosa* ATCC 25922	4.00	-	0.4	-	-	-	
Ethanol (µg/mL)	Disc dilution & Broth microdilution	*Escherichia coli* ESβL(+) (CI)	312	312	0.0312	0.0312	1 *	20	[29]
		*Klebsiella pneumoniae* ESβL(+) (CI)	625	625	0.0625	0.0625	1 *	20	
		*Klebsiella pneumoniae* CRE(+) 1 (CI)	312	312	0.0312	0.0312	1 *	21	
		*Klebsiella pneumoniae* CRE(+) 2 (CI)	312	312	0.0312	0.0312	1 *	24	
		*Klebsiella pneumoniae* CRE(+) 3 (CI)	625	625	0.0625	0.0625	1 *	23	
		*Klebsiella pneumoniae* CRE(+) 4 (CI)	312	312	0.0312	0.0312	1 *	23	
		*Serratia marcescens* CRE(+) (CI)	312	312	0.0312	0.0312	1 *	20	
		*Pseudomonas aeruginosa* MβL(+) 1 (CI)	312	312	0.0312	0.0312	1 *	17	
		*Pseudomonas aeruginosa* MβL(+) 2 (CI)	312	312	0.0312	0.0312	1 *	19	
		*Pseudomonas aeruginosa* MβL(+) 3 (CI)	156	156	0.0156	0.0156	1*	28	
		*Acinetobacter baumannii* MβL(+) 1 (CI)	625	625	0.0625	0.0625	2 *	23	
		*Acinetobacter baumannii* MβL(+) 2 (CI)	156	312	0.0156	0.0312	2 *	24	
		*Acinetobacter baumannii* MβL(+) 3 (CI)	312	312	0.0312	0.0312	1 *	24	
		*Acinetobacter baumannii* MβL(+) 4 (CI)	312	312	0.0312	0.0312	1 *	23	
		*Acinetobacter baumannii* MβL(+) 5 (CI)	625	625	0.0625	0.0625	1 *	26	
Methanol (µg/mL)	Disc dilution & Broth microdilution	*Escherichia coli* ESBL(+) (CI)	312	312	0.0312	0.0312	1 *	19	[29]
		*Klebsiella pneumoniae* ESβL(+) (CI)	625	625	0.0625	0.0625	1 *	19	
		*Klebsiella pneumoniae* CRE(+) 1 (CI)	625	625	0.0625	0.0625	1 *	21	
		*Klebsiella pneumoniae* CRE(+) 2 (CI)	312	312	0.0312	0.0312	1 *	23	
		*Klebsiella pneumoniae* CRE(+) 3 (CI)	625	625	0.0625	0.0625	1 *	22	
		*Klebsiella pneumoniae* CRE(+) 4 (CI)	312	312	0.0312	0.0312	1 *	22	
		*Serratia marcescens* CRE(+) (CI)	312	312	0.0312	0.0312	1 *	19	
		*Pseudomonas aeruginosa* MβL(+) 1 (CI)	625	625	0.0625	0.0625	1 *	15	
		*Pseudomonas aeruginosa* MβL(+) 2 (CI)	625	625	0.0625	0.0625	1 *	18	
		*Pseudomonas aeruginosa* MβL(+) 3 (CI)	156	156	0.0156	0.0156	1 *	27	
		*Acinetobacter baumannii* MβL(+) 1 (CI)	625	625	0.0625	0.0625	1 *	22	
		*Acinetobacter baumannii* MβL(+) 2 (CI)	625	1250	0.0625	0.125	2 *	24	
		*Acinetobacter baumannii* MβL(+) 3 (CI)	625	625	0.0625	0.0625	1 *	23	
		*Acinetobacter baumannii* MβL(+) 4 (CI)	312	312	0.0312	0.0312	1 *	22	
		*Acinetobacter baumannii* MβL(+) 5 (CI)	625	625	0.0625	0.0625	1 *	25	
SC-CO_2_ 15MPa (µg/mL)	Disc dilution & Broth microdilution	*Escherichia coli* ESβL(+) (CI)	625	1250	0.0625	0.125	2 *	15	[29]
		*Klebsiella pneumoniae* ESβL(+) (CI)	1250	1250	0.125	0.125	1 *	15	
		*Klebsiella pneumoniae* CRE(+) 1 (CI)	625	625	0.0625	0.0625	1 *	15	
		*Klebsiella pneumoniae* CRE(+) 2 (CI)	625	1250	0.0625	0.125	2 *	20	
		*Klebsiella pneumoniae* CRE(+) 3 (CI)	625	1250	0.0625	0.125	2 *	16	
		*Klebsiella pneumoniae* CRE(+) 4 (CI)	625	625	0.0625	0.0625	1 *	16	
		*Serratia marcescens* CRE(+) (CI)	312	312	0.0312	0.0312	1 *	18	
		*Pseudomonas aeruginosa* MβL(+) 1 (CI)	1250	1250	0.125	0.125	1 *	11	
		*Pseudomonas aeruginosa* MβL(+) 2 (CI)	1250	1250	0.125	0.125	1 *	14	
		*Pseudomonas aeruginosa* MβL(+) 3 (CI)	625	625	0.0625	0.0625	1 *	12	
		*Acinetobacter baumannii* MβL(+) 1 (CI)	625	625	0.0625	0.0625	1 *	20	
		*Acinetobacter baumannii* MβL(+) 2 (CI)	625	1250	0.0625	0.125	2 *	20	
		*Acinetobacter baumannii* MβL(+) 3 (CI)	625	625	0.0625	0.0625	1 *	19	
		*Acinetobacter baumannii* MβL(+) 4 (CI)	625	625	0.0625	0.0625	1 *	18	
		*Acinetobacter baumannii* MβL(+) 5 (CI)	625	625	0.0625	0.0625	1 *	21	
SC-CO_2_ 20MPa (µg/mL)	Disc dilution & Broth microdilution	*Escherichia coli* ESβL(+) (CI)	625	625	0.0625	0.0625	1 *	16	[29]
		*Klebsiella pneumoniae* ESβL(+) (CI)	625	625	0.0625	0.0625	1 *	16	
		*Klebsiella pneumoniae* CRE(+) 1 (CI)	312	312	0.0312	0.0312	1 *	16	
		*Klebsiella pneumoniae* CRE(+) 2 (CI)	312	625	0.0312	0.0625	2 *	20	
		*Klebsiella pneumoniae* CRE(+) 3 (CI)	625	625	0.0625	0.0625	2 *	17	
		*Klebsiella pneumoniae* CRE(+) 4 (CI)	312	312	0.0312	0.0312	1 *	17	
		*Serratia marcescens* CRE(+) (CI)	312	312	0.0312	0.0312	1 *	18	
		*Pseudomonas aeruginosa* MβL(+) 1 (CI)	625	625	0.0625	0.0625	1 *	11	
		*Pseudomonas aeruginosa* MβL(+) 2 (CI)	625	625	0.0625	0.0625	1 *	15	
		*Pseudomonas aeruginosa* MβL(+) 3 (CI)	625	625	0.0625	0.0625	1 *	14	
		*Acinetobacter baumannii* MβL(+) 1 (CI)	625	625	0.0625	0.0625	1 *	22	
		*Acinetobacter baumannii* MβL(+) 2 (CI)	312	625	0.312	0.0625	2 *	22	
		*Acinetobacter baumannii* MβL(+) 3 (CI)	625	625	0.0625	0.0625	1 *	22	
		*Acinetobacter baumannii* MβL(+) 4 (CI)	312	312	0.312	0.312	1 *	21	
		*Acinetobacter baumannii* MβL(+) 5 (CI)	625	625	0.0625	0.0625	1 *	24	
Ethanol (mg/mL)	Agar well diffusion & Broth microdilution	*Aggregatibacter actino-mycetemcomitans* ATCC 33384	1.04	2.08	0.104	0.208	2 *	≥20	[17]
		*Fusobacterium nucleatum* ATCC 25586	1.30	2.08	0.13	0.208	1.6 *	≥20	
Ethyl acetate	Broth dilution	*Vibrio harveyi*	1600	-	0.16	-	-	-	[32]
Extract-Ag nanoparticles	Kirby-Bauer’s Disc diffusion	*Pseudomenas aeruginosa* ATCC 27853	-	-	-	-	-	21.95 ± 0.45	[25]
		*Salmonella typhi* ATCC 14028	-	-	-	-	-	29.55 ± 0.45	
		*Escherichia coli* ATCC 25922	-	-	-	-	-	27.12 ± 0.38	
Extract-CaO nanoparticles	Agar well diffusion	*Escherichia coli* ATCC 25922	-	-	-	-	-	18	[28]
		*Pseudomonas aeruginosa* ATCC 27853	-	-	-	-	-	13	
BLEO-nanoemulsion (µL/mL)	Microdilution plate	*Escherichia coli* MTCC 443	0.5–1	1–1.5	0.05–0.1	0.1–0.15	1–3 *	-	[26]
		*Klebsiella pneumoniae* MTCC 432	1–1.25	2–2.5	0.1–0.125	0.2–0.25	1–2 *	-	
		*Pseudomonas aeruginosa* MTCC 424	0.5–0.75	1–1.5 µL/mL	0.05–0.075	0.1–0.15	2 *	-	
BLEO (mg/mL)	Micro-dilution broth & growth inhibitory assay	*Acinetobacter baumannii* (CI)	8	8	0.8	0.8	1 *	-	[24]
		*Escherichia coli ATCC* 25922	0.3	0.3	0.03	0.03	1 *	-	
		*Escherichia coli* (CI)	2	2	0.2	0.2	1 *	-	
		*Klebsiella pneumoniae* (CI)	4	4	0.4	0.4	1 *	-	
		*Pseudomonas aeruginosa* ATCC 27853	0.5	0.5	0.05	0.05	1 *	-	
		*Pseudomonas aeruginosa* (CI)	2	2	0.2	0.2	1 *	-	
		*Proteus vulgaris* (CI)	4	4	0.4	0.4	1 *	-	
BLEO + Gentamicin (mg/mL)	Micro-dilution broth & growth inhibitory assay	*Escherichia coli ATCC* 25922	0.5-1	-	0.05–0.1	-	-	-	[24]

BLEO = betel leaves essential oil, ESβL = Extended spectrum β-lactamase, MRSA = Methicillin-resistant *Staphylococcus aureus*, MβL = metallo-β-lactam, - = data not available, * = bactericidal

**Table 3:** *Piper betle* against Gram-positive bacteria.

Extract/Preparation/Isolate (Unit for Activities)	Method	BACTERIA SPECIES	Activitites		Recalculated (%)		MBC/MIC	Inhibition Zone (mm)	Reference
			MIC	MBC	MIC	MBC			
Ethanol	Agar well diffusion	*Bacillus subtilis*	-	-	-	-	-	13.2–15.8	[11]
		*Staphylococcus aureus*	-	-	-	-	-	9.7–18.0	
		*Micrococcus luteus*	-	-	-	-	-	5.0–5.4	
Water	Agar well diffusion	*Bacillus subtilis*	-	-	-	-	-	4.9–6.8	[11]
		*Staphylococcus aureus*	-	-	-	-	-	5.4–12.3	
		*Micrococcus luteus*	-	-	-	-	–	3.5–4.2	
Ethanol (µg/mL)	Disk diffusion	*Staphylococcus aureus* ATCC 29223	312	312	0.0312	0.0312	1 *	30	[27]
		MRSA #1 (CI)	156	312	0.0156	0.0312	2 *	32	
		MRSA #2 (CI)	156	156	0.0156	0.0156	1 *	34	
		MRSA #3 (CI)	156	156	0.0156	0.0156	1 *	28	
		MRSA #4 (CI)	78	78	0.0078	0.0078	1 *	34	
		VRE	19	19	0.0019	0.0019	1 *	28	
Ethyl acetate (µg/µL)	Broth microdilution	*Staphylococcus aureus* ATCC 25923	0.50	-	0.0005	-	-	-	[12]
		*Propionibacterium acnes* ATCC 6919	2.00	-	0.002	-	-	-	
		*Staphylococcus epidermidis* ATCC 12228	4.00	-	0.004	-	-	-	
		*Streptococcus pyogenes* ATCC 19615	4.00	-	0.004	-	-	-	
Acetone (µg/µL)	Broth microdilution	*Staphylococcus aureus* ATCC 25923	0.25	-	0.00025	-	-	-	[12]
		*Propionibacterium acnes* ATCC 6919	2.00	-	0.002	-	-	-	
		*Staphylococcus epidermidis* ATCC 12228	4.00	-	0.004	-	-	-	
		*Streptococcus pyogenes* ATCC 19615	4.00	-	0.004	-	-	-	
Dichloromethane (µg/µL)	Broth microdilution	*Staphylococcus aureus* ATCC 25923	1.00	-	0.001	-	-	-	[12]
		*Propionibacterium acnes* ATCC 6919	4.00	-	0.004	-	-	-	
		*Staphylococcus epidermidis* ATCC 12228	4.00	-	0.004	-	-	-	
		*Streptococcus pyogenes* ATCC 19615	4.00	-	0.004	-	-	-	
Ethanol (µg/mL)	Disk diffusion	MRSA 1–7	78–156	78–312	0.0078–0.0156	0.0078–0.0312	1–2 *	28–3833	[29]
		VRE 1–3	19–156	19–156	0.0019–0.0156	0.0019–0.0156	1 *	25–3228	
Methanol (µg/mL)	Disk diffusion	MRSA 1–7	78–312	78–312	0.0078–0.0312	0.0078–0.0312	1–2 *	28–3432	[29]
		VRE 1–3	19–156 µg/mL19µg/mL	19–156 µg/mL19µg/mL	0.0019–0.0156	0.0019–0.0156	1 *	25–3226	
SC-CO_2_ 15MPa (µg/mL)	Disk diffusion	MRSA 1–7	312–625	312–1250	0.0312–0.0625	0.0312–0.125	1 *	21–3025	[29]
		VRE 1–3	19–156	19–156	0.0019–0.0156	0.0019–0.0156	1 *	15–2820	
SC-CO_2_ 20MPa (µg/mL)	Disk diffusion	MRSA 1–7	156–625	156–625	0.0156–0.0625	0.0156–0.0625	1 *	22–3325	[29]
		VRE 1–3	19–156	19–156	0.0019–0.0156	0.0019–0.0156	1 *	15–3124	
Ethanol (mg/mL)	Agar well diffusion & Broth microdilution	*Enterobacter faecalis* ATCC 19433	5.21	8.33	0.521	0.833	1.6 *	10–20	[17]
		*Lactobacillus fermentum* ATCC 14931	4.17	8.33	0.417	0.833	2 *	10–20	
		*Lactobacillus salivarius* ATCC 11741	4.17	8.33	0.417	0.833	2 *	10–20	
		*Streptococcus sobrinus* ATCC 33478	1.56	3.17	0.156	0.317	2 *	≥20	
		*Streptococcus mutans* ATCC 25175	1.56	3.17	0.156	0.317	2 *	≥20	
Hexane (µg/mL)	Disk diffusion	*Streptococcus gordonii* DMST 38731	1.00	2.00	0.0001	0.0002	2 *	8.00 ± 0.00	[30]
		*Streptococcus mutans* DMST 18777	2.00	2.00	0.0002	0.0002	1 *	-	
Ethyl acetate (µg/mL)		*Streptococcus gordonii* DMST 38731	0.50	2.00	0.00005	0.0002	4 **	12.50 ± 0.70	[30]
		*Streptococcus mutans* DMST 18777	1.00	2.00	0.0001	0.0002	2 *	11.00 ± 0.00	
Ethanol	Agar well diffusion	*Staphylococcus aureus* (CI)	-	-	-	-	-	2..500–20.375	[37]
Extract-Ag nanoparticles	Kirby-Bauer’s Disc diffusion	*Staphylococcus aureus* ATCC 25923	-	-	-	-	-	32.78 ± 0.64	[25]
Extract-CaO nanoparticles	Agar well diffusion	*Staphylococcus aureus* ATCC 25923	-	-	-	-	-	13	[28]
		*Streptococcus mutans* MTCC 890	-	-	-	-	-	12	
BLEO-nanoemulsion (µL/mL)	Microdilution plate	*Staphylococcus aureus* MTCC 1144	0.5–0.75	1–1.5	0.05–0.075	0.1–0.15	2 *		[26]
		*Bacillus cereus* MTCC 1272	0.5–0.75	0.75–1.5	0.05–0.075	0.1–0.15	2 *	-	
BLEO (mg/mL)	Micro-dilution broth & growth inhibitory assay	*Escherichia faecalis* (CI)	4	4	0.4	0.4	1 *		[24]
		*Propionibacterium acnes* ATCC 6919	1	1	0.1	0.1	1 *	-	
		*Staphylococcus aureus* ATCC 25923	0.5	0.5	0.05	0.05	1 *	-	
		*Staphylococcus epidermidis* ATCC 12228	0.5	0.5	0.05	0.05	1 *	-	
		*Streptococcus peroris* (CI)	2	2	0.2	0.2	1 *	-	
		MRSA (CI)	8	8	0.8	0.8	1 *	-	
BLEO+Gentamicin (mg/mL)	Micro-dilution broth & growth inhibitory assay	*Staphylococcus epidermidis* ATCC 12228	1-2	-	0.1–0.2		-	-	[24]
Allylpyrocatechols I (µg/mL)	Kirby–Bauer disk diffusion	*Streptococcus sanguinis* ATCC 10566	39.1	78.1	0.00391	0.00781	2 *	11.85–25.15	[15]
4-allylpyrocatechol (µg/mL)	Broth microdilution	*Streptococcus intermedius* DMST 42700	200	500	0.02	0.05	2.5 *	-	[35]
		*Streptococcus mutans* DMST 41283	200	500	0.02	0.05	2.5 *	-	

BLEO = betel leaves essential oil, CI = Clinical isolate, MRSA = Methicillin-resistant *Staphylococcus aureus*, VRE = vancomycin-resistant *Enterococcus*, - = data not available, * = bactericidal, ** = bacteriostatic.

**Table 4:** *Piper betle* against various fungal species.

Extract/Preparation/Isolate (Unit for Activities)	Method	Fungal Species	Activities		Recalculated (%)		MFC/MIC	Inhibition Zone (mm)	Reference
			MIC	MFC	MIC	MFC			
Young leaves									[39]
Ethanol (μg/mL)	Broth microdilution	*Candida albicans* (CI)	500	-	0.05	-	-	8–15	
Ethyl acetate (μg/mL)	Broth microdilution	*Candida albicans* (CI)	250	-	0.025	-	-	10–22	
Mature leaves									[39]
Ethanol (μg/mL)	Broth microdilution	*Candida albicans* (CI)	750	-	0.075	-	-	5–22	
Ethyl acetate (μg/mL)	Broth microdilution	*Candida albicans* (CI)	125	-	0.0125	-	-	17–26	
Ethyl acetate	Well-diffusion	*Aspergillus niger*	-	-	-	-	-	28	[10]
		*Aspergillus sp.*	-	-	-	-	-	5	
Hexane	Well-diffusion	*Aspergillus niger*	-	-	-	-	-	28	[10]
		*Aspergillus sp.*	-	-	-	-	-	8	
Hexane (mg/mL)	Disk diffusion	*Candida albicans* DMST 8684	1.00	2.00	0.1	0.2	2 *	21.00 ± 1.40	[30]
		*Candida albicans* DMST 5815	1.00	4.00	0.1	0.4	4 **	20.67 ± 0.58	
Ethyl acetate (mg/mL)	Disk diffusion	*Candida albicans* DMST 8684	0.50	2.00	0.05	0.2	4 **	23.00 ± 0.00	[30]
		*Candida albicans* DMST 5815	1.00	2.00	0.1	0.2	2 *	24.33 ± 0.58	
BLEO (μL/mL)	Solid dilution	*Alternaria alternate*	0.53	-	0.053	-	-	-	[18]
		*Aspergillus candidus*	0.57	-	0.057	-	-	-	
		*Aspergillus flavus*	0.7	-	0.07	-	-	-	
		*Aspergillus fumigatus*	0.40	-	0.04	-	-	-	
		*Aspergillus niger*	0.73	-	0.073	-	-	-	
		*Aspergillus sydowi*	0.63	-	0.063	-	-	-	
		*Aspergillus terreus*	0.60	-	0.060	-	-	-	
		*Cladosporium cladosporoides*	0.67	-	0.067	-	-	-	
		*Culcularia lunata*	0.50	-	0.05	-	-	-	
		*Fusarium oxysporum*	0.50	-	0.05	-	-	-	
		*Mucor sp.*	0.37	-	0.037	-	-	-	
		*Mycelia sterilia*	0.30	-	0.03	-	-	-	
		*Nugrospora sp.*	0.53	-	0.053	-	-	-	
		*Penicillium italicum*	0.40	-	0.04	-	-	-	
BLEO (mg/mL)	Microdilution broth & growth inhibitory assay	*Aspergillus niger* ATCC 16404	2	2	0.2	0.2	1 *	-	[24]
		*Candida albicans* ATCC 10231	1.5	1.5	0.15	0.15	1 *	-	
		*Candida albicans* (CI)	2	2	0.2	0.2	1 *	-	
		*Candida tropicalis* ATCC 750	2	2	0.2	0.2	1 *	-	
BLEO (μL/mL)	Broth microdilution	*Trichophyton mentagrophytes* (CI)	0.2–0.4	0.4	0.00002–0.00004	0.00004	1–2 *	-	[40]
		*Trichophyton mentagrophytes* DMST 19735	0.2–0.4	0.4	0.00002–0.00004	0.00004	1–2 *	-	
		*Microsporum canis* (CI)	0.2–0.4	0.4	0.00002–0.00004	0.00004	1–2 *	-	
		*Microsporum canis* DMST 29297	0.2	0.4	0.00002–0.00004	0.00004	2 *	-	
		*Microsporum gypseum* (CI)	0.4–0.8	0.8	0.00004–0.00008	0.00008	1–2 *	-	
		*Microsporum gypseum* DMST 21146	0.8	0.8	0.00008	0.00008	1 *	-	
BLEO (%*v*/*v*)	Disk diffusion	*Candida albicans* ATCC 10231	0.078	-	0.078	-	-	33.83 + 0.76	[2]
		*Candida glabrata* ATCC 90030	0.039	-	0.039	-	-	33.83 + 0.76	
		*Candida krusei* ATCC 6258	0.078	-	0.078	-	-	32.66 + 0.57	
		*Candida parapsilosis* ATCC 22019	0.039	-	0.039	-	-	33.83 + 0.76	
		*Candida pseudotropicalis* (CI)	0.039	-	0.039	-	-	33.50+0.50	
		*Candida stellatoidia* (CI)	0.039	-	0.039	-	-	35.50+0.86	
		*Candida tropicalis* (CI)	0.078	-	0.078	-	-	30.83+0.28	
BLEO-microemulsion (μL/mL)	Broth dilution	*Aspergillus flavus*	-	15	-	1.5	-	-	[41]
		*Penicillium expansum*	-	15	-	1.5	-	-	
Hydroxychavicol (μg/mL)	Broth microdilution	*Aspergillus flavus* MTCC 1973, 2799	250	250	0.025	0.025	1 *	-	[38]
		*Aspergillus flavus* (CI)	125-500	125–500	0.0125–0.05	0.0125–0.05	1 *		
		*Aspergillus fumigatus* MTCC 1811	250	250	0.025	0.025	1 *	-	
		*Aspergillus niger* ATCC 16404	125	125	0.0125	0.0125	1 *	-	
		*Aspergillus niger* (CI)	125-250	125-250	0.0125–0.05	0.0125–0.05	1 *		
		*Aspergillus parasiticus* MTCC 2796	250	250	0.025	0.025	1 *	-	
		*Candida albicans* ATCC 90028, 10231	250	250	0.025	0.025	1 *	-	
		*Candida albicans* (CI)	125–500	250–500	0.0125–0.05	0.0125–0.05	1–2 *		
		*Candida glabrata* ATCC 90030	31.25	31.25	0.003125	0.003125	1 *	-	
		*Candida glabrata* (CI)	15.62–31.25	15.62–62.5	0.001562–0.003125	0.001562–0.00625	1–2 *		
		*Candida krusei* ATCC 22019	15.62	62.5	0.001562	0.00625	4 **	-	
		*Candida krusei* (CI)	15.62–31.25	15.62–31.25	0.001562–0.003125	0.001562–0.003125	1 *	-	
		*Candida neoformans* ATCC 204092	62.5	62.5	0.00625	0.00625	1 *	-	
		*Candida neoformans* (CI)	62.5	62.5	0.00625	0.00625	1 *	-	
		*Candida parapsilosis* ATCC 22019	31.25	31.25	0.003125	0.003125	1 *	-	
		*Candida parapsilosis* (CI)	31,25–62.5	31,25–62.5	0.003125–0.00625	0.003125–0.00625	1 *	-	
		*Candida tropicallis* ATCC 750	250	250	0.025	0.025	1 *	-	
		*Candida tropicallis* (CI)	125–500	250–500	0.0125–0.05	0.025–0.05	1–2 *	-	
		*Epidermophyton floccosum* MTCC 613	15.62	15.62	0.001562	0.001562	1 *	-	
		*Epidermophyton floccosum* (CI)	15.62	31.25	0.001562	0.003125	2 *		
		*Microsporum canis* MTCC 2820	15.62	31.25	0.001562	0.003125	2 *	-	
		*Microsporum canis* (CI)	15.62	31.25	0.001562	0.003125	2 *	-	
		*Micosporum gypsium* MTCC 2819	15.62	31.25	0.001562	0.003125	2 *	-	
		*Micosporum gypsium* (CI)	7.81–15.62	15.62–31.25	0.000781–0.001562	0.001562–0.003125	2 *	-	
		*Trichophyton mentagrophytes* ATCC 9533	15.62	15.62	0.001562	0.001562	1 *	-	
		*Trichophyton mentagrophytes* (CI)	15.62–31.25	15.62–62.5	0.001562–0.003125	0.001562–0.00625	1–2 *	-	
		*Trichophyton rubrum MTCC* 296	31.25	31.25	0.003125	0.003125	1 *	-	
		*Trichophyton rubrum* (CI)	15.62–62.5	31.25–62.5	0.001562–0.00625	0.003125–0.00625	1–2 *	-	
4-allylpyrocatechol (μg/mL)	Broth Microdilution	*Candida albicans* DMST 8684	400	500	0.04	0.05	1.25 *	-	[35]

BLEO = betel leaves essential oil, CI = clinical isolate, MIC = minimum inhibitory concentration; MFC = minimum fungicidal concentration, - = Data not available, * = fungicidal, ** = fungistatic.

## Data Availability

Not applicable.
